# Scoping Review of Extraction Methods for Detecting β-Lactam Antibiotics in Food Products of Animal Origin

**DOI:** 10.3390/molecules30091937

**Published:** 2025-04-27

**Authors:** Joanna Pacyńska, Przemysław Niedzielski

**Affiliations:** 1 Department of Analytical Chemistry, Faculty of Chemistry, Adam Mickiewicz University in Poznań, 8 Uniwersytetu Poznańskiego Street, 61-614 Poznań, Poland; joanna.pacynska@amu.edu.pl; 2 Provincial Veterinary Inspectorate in Poznań, 250 Grunwaldzka Street, 60-166 Poznań, Poland

**Keywords:** β-lactam antibiotics, extraction methods, food safety, detection techniques, animal products, antibiotic residues

## Abstract

The widespread use of β-lactam antibiotics in veterinary medicine and food production has contributed to the rise of antibiotic-resistant bacteria, posing significant health risks to humans. This issue is recognized by various regulatory agencies, which settled maximum residue limits (MRLs) for antibiotics in animal-derived foods. To adhere to these regulations, sensitive and selective methods are required for monitoring antibiotic residues. Due to the critical importance of sample preparation in the analysis, numerous extraction techniques have been developed. This review focuses on various methodologies for extracting β-lactam antibiotics from different food matrices. The paper summarizes the procedures for the extraction of β-lactam antibiotics identified in the literature, indicating their detailed methodology. The summary may be useful for any laboratories preparing new applications for the determination of antibiotics in food. Research studies analyzed in the paper were collected from databases, such as Google Scholar, PubMed, and Scopus. After a close evaluation of about 200 articles (published between 2010 and 2024), 35 of them, which met the criteria, were included in the analysis.

## 1. Introduction

The term “antibiotics” is used to refer to drugs of natural, semisynthetic, or synthetic origin [1]. These drugs are widely used in clinical practice, since the discovery of penicillin. The purpose of their use on bodies is to kill or stop the growth of particular microorganisms. Antibiotics are commonly used in human as well as in veterinary medicine to treat infectious diseases. As international trade continues to expand, including the trade of meat and other animal-based foods, antibiotics are increasingly used in animal feed to enhance growth and support health [2].

However, the extensive use of antibiotics in veterinary medicine and meat production may be a cause of concern [3]. The frequent use of the antimicrobial is seen as the main cause of antibiotic residue in animals’ meat. Arguably, food is the main source of antibiotics that are stored in human bodies. The consumption of meat with excess antibiotics can have an impact on humans’ health, especially on creating antibiotic resistance. In addition to promoting antimicrobial resistance, the presence of antibiotic residues in food can cause alterations in the human gut microbiota, leading to dysbiosis, weakened immunity, and increased susceptibility to infections. Furthermore, antibiotic residues may trigger allergic reactions, particularly in sensitive individuals. Some antibiotic residues are also associated with immunopathological effects, nephrotoxicity, hepatotoxicity, reproductive disorders, and, in rare cases, mutagenic or carcinogenic effects. Therefore, the presence of antibiotic residues in food represents a serious public health risk [4]. This resistance may be transmitted to the general population, creating antibiotic-resistant diseases [5]. What is more, the effects of using extensive amounts of drugs in animal-derived food production may be very far-reaching, as their residues might be transferred into communal wastewater through urine [6].

The risk of creating antibiotic-resistant diseases, as well as the increasing consumers’ awareness of food safety, led to developing food and nutrition safety regulations. Professionals in the field are obliged to control and ensure access to good quality and nutritious food [7]. The main purpose of those regulations is to supply humans with food that is free from any contamination that could be harmful to their health.

For enforcement at the national level and in the international food trade, residues of antibiotics and other veterinary drugs are now monitored around the world by many governments and private laboratories. However, differences exist in the regulation of antibiotics for food-producing animals between countries. For example, in the United States of America, the Federal Food, Drug, and Cosmetic Act was amended in 1996 by the Food and Drug Administration Center for Veterinary Medicine to strengthen control over veterinary drugs. Instead of banning all antibiotics, the United States establishes maximum residue limits (referred to as “tolerances”) for many substances to ensure food safety, which are published in 21 CFR Part 556. In the European Union, the 27 Member States amended Directive 2001/82/EC and Regulation 726/2004 of the European Parliament and the Council. In the EU, the use of antibiotics is more strictly regulated, and maximum residue limits (MRLs) for veterinary drugs in food are clearly defined under Regulation (EU) No 37/2010. This means that while both the United States and the European Union aim to protect public health, their approaches to antibiotic regulation differ, particularly in how they set and enforce limits for residues in food products [8].

Although the use of antibiotics in meat production is almost inevitable, the European Union formulated standards that determine the maximum residue limits (MRLs) of veterinary drugs in food of animal origin. The limits for antibiotic residues have been established by Commission Regulation (EU) 37/2010 and apply to various products of animal origin [2,9]. This regulation sets the maximum allowable levels of veterinary drug residues in animal tissues following the administration of veterinary medicines. It is important to note that MRLs differ depending on the type of antibiotic, the type of animal, and even the type of tissue. Each antibiotic is assigned specific MRLs, indicating the maximum residue level that is considered safe in a particular animal product for consumers. Adhering to MRLs is crucial to minimize health risks and to control antibiotic resistance. International and national organizations monitor compliance with these standards through food control programs to ensure the safety of animal-origin food products on the market.

Considering these regulations and the need for reliable monitoring of β-lactam antibiotic residues in food, this review examines various extraction and detection methods. The first section provides a general overview of β-lactam antibiotics, followed by a discussion of extraction methods used in the analysis of antibiotic residues. Earlier studies used traditional extraction techniques for β-lactam antibiotics, whereas in recent years (2010–2024) there has been significant progress in both extraction methods, such as QuEChERS, and detection methods, including the widespread use of high-performance liquid chromatography coupled with tandem mass spectrometry (LC-MS/MS), allowing for the simultaneous determination of multiple β-lactams in complex food matrices. In the final section, specific examples of extraction methods described in the literature are presented in chronological order, from the oldest to the most recent developments.

## 2. Methodology

Research studies focusing on the detection of antibiotic residues were collected from the Google Scholar, PubMed, and Scopus databases. The research concentrates on publications addressing the topic of extraction of β-lactam antibiotic residues from animal-derived food and their chromatographic analysis from 2010 to 2024. There are many methods for antibiotic analysis, so a selection of publications was made to ensure a variety of extraction and analytical techniques. The following keywords were used in the search: “antibiotic”, “β-lactam”, “antibiotic residues”, “β-lactam antibiotic residues”, “extraction of antibiotics from food”, and “drug residues in animal products”. Over 600 records were identified, and after removing duplicates and irrelevant entries, approximately 200 articles were selected for further evaluation. Publications addressing the extraction of β-lactam antibiotic residues from animal-derived food and their chromatographic analysis were included. Only peer-reviewed studies published in international journals, using chromatographic techniques (e.g., HPLC, LC-MS/MS, and UHPLC), with full-text availability were selected. Based on title and abstract screening, studies focusing on microbiological and immunochemical methods, those without chromatographic detection, and those using unsuitable matrices (e.g., blood, water, and drugs) were excluded. Additionally, review articles, studies not related to animal-derived food matrices, and purely in vitro experiments without food matrices were also excluded. Some full texts were unavailable, and additional studies were removed due to missing data or being published in languages other than English. Ultimately, 35 studies meeting the criteria were included in the review, providing insights into β-lactam antibiotic extraction methods from animal-derived food and their chromatographic analysis.

## 3. β-Lactam Antibiotics

There are a few types of antibiotics certified by license to be used in animals’ treatment. Antibiotics used in veterinary medicine can be categorized into certain groups depending on their chemical structure or action mechanism [10]. The amount of each of the drugs must be controlled individually but all of the antibiotics should be dosed under veterinary supervision.

The authors decided to focus on β-lactam antibiotics due to their extensive use in animal-derived food, as well as their efficacy and consequently their relevance in food safety. β-Lactams are among the most widely used classes of antibiotics in veterinary medicine because of their broad-spectrum activity, relatively low toxicity, and cost-effectiveness. However, their frequent application has contributed significantly to the emergence of antimicrobial resistance, posing a serious threat to public health. Monitoring β-lactam residues in food is, therefore, essential to ensure consumer safety and to prevent the spread of resistant bacteria through the food chain.

The main negative effect of β-lactam use is acute allergic reaction, which is rare and mainly associated with penicillins. However, adverse effects, such as urticaria or angioneurotic edema, were observed more commonly. There were instances of lethargy, pyrexia, vomiting, and other reactions in pigs and horses. Sensitization as well as hypersensitivity to penicillin are quite common in humans during treatment [10].

**Figure 1:**
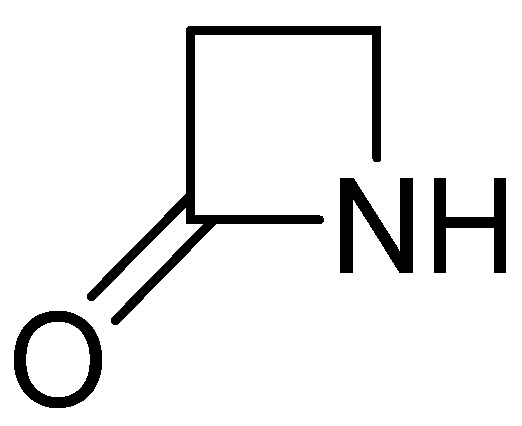
The β-lactam ring.

**Figure 2:**
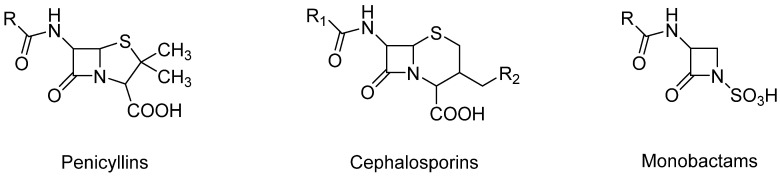
Chemical structures of β-lactams.

The review examines the antibiotics’ usage, extraction methods, and detection techniques to ensure food safety and regulatory compliance. β-Lactams create a group of natural or semisynthetic antibiotics that contain a β-lactam ring in their molecular structure (Figure 1 and Figure 2) [11]. Their action mechanism is based on restraining the synthesis of the bacterial cell wall, causing their death. This group contains drugs such as penicillin derivatives, cephalosporins, monobactams, carbapenems, and β-lactamase inhibitors [12]. They are the biggest as well as the most widely utilized antibiotics in clinical practice. In veterinary medicine, β-lactam antibiotics are used to treat numerous bacterial infections in various animals, with pets, such as dogs or cats, and livestock, such as cattle, pigs, and poultry, as examples [13].

**Figure 3:**
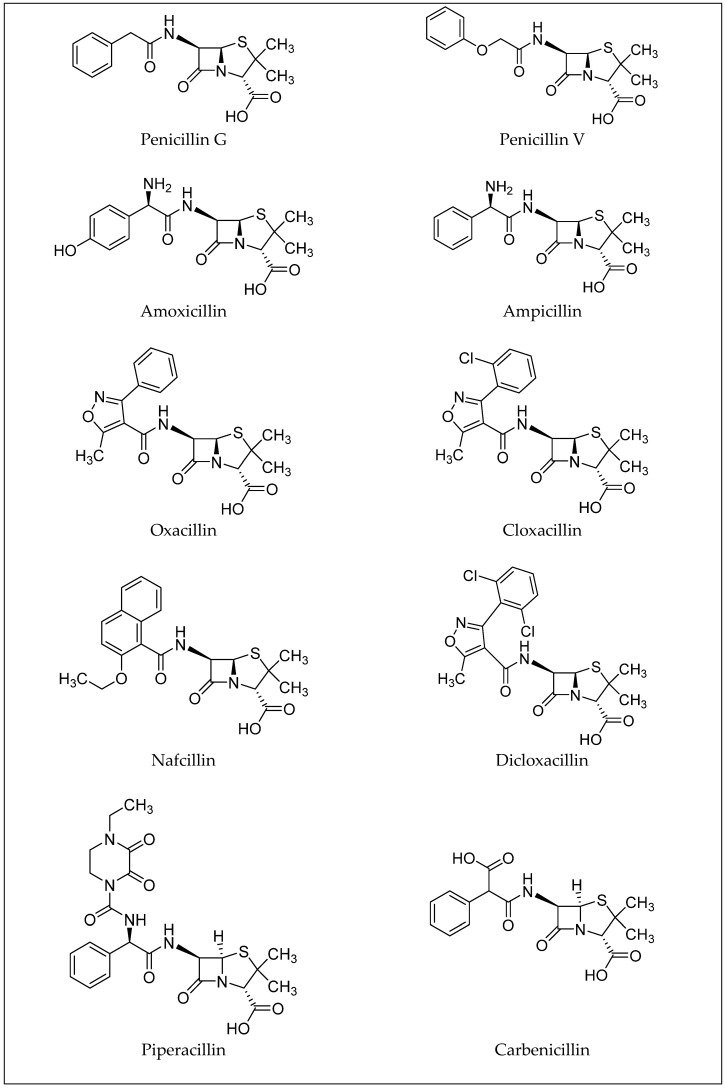
Examples of antibiotics from the penicillin group.

Penicillins, a subgroup of beta-lactam antibiotics, are derived from both semisynthetic and natural sources and share a feature concerning their structure: the aminopenicillanic acid nucleus. This nucleus consists of a beta-lactam ring attached to a thiazolidine ring (Figure 2). Natural penicillins are produced by various species of the *Penicillium* fungi. The diversity in the penicillin class is due to alterations at position 6 of the aminopenicillanic acid ring, with variations in the side chain leading to differences in antibacterial activity and pharmacokinetic properties [14]. These modifications allow for the classification of penicillins into several types: natural penicillins (penicillins G and V), penicillins resistant to staphylococcal penicillinase (oxacillin, dicloxacillin, and nafcillin), aminopenicillin (amoxicillin and ampicillin), carboxypenicillin (carbenicillin), and ureidopenicillin (piperacillin) [6]. Examples of antibiotics from the penicillin group are shown in Figure 3. Toxicological data, including molecular formulas, CAS numbers, LD50 values, and PubChem references for the antibiotics shown, are summarized in Table 1 [15,16,17,18,19,20,21,22,23,24].

**Figure 4:**
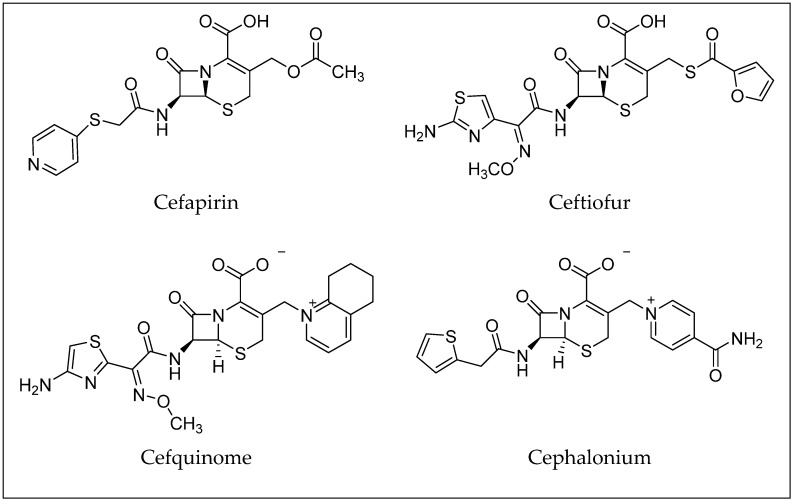

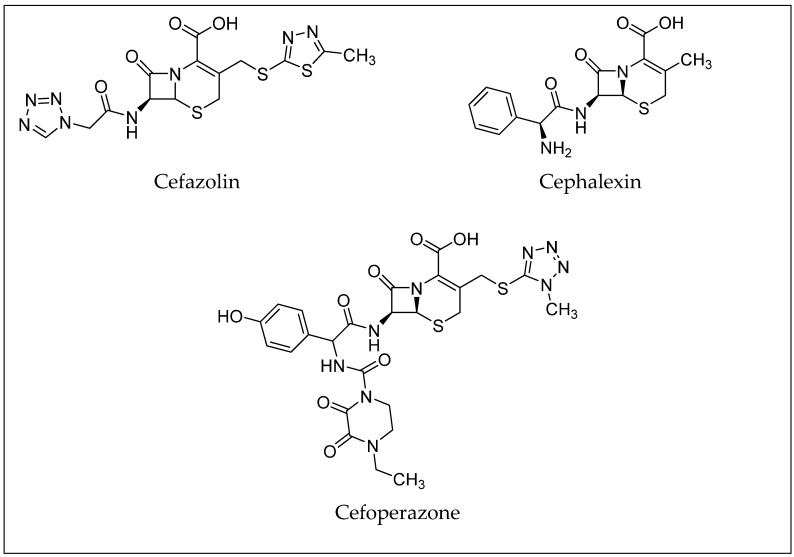
Examples of antibiotics from the cephalosporin group.

Cephalosporins constitute a significant group of antibiotics that are structurally and functionally related to penicillins but with a broader spectrum of activity. Cephalosporins are categorized by generations, which generally correspond to their order of development and spectrum of antibacterial activity [25]. First-generation cephalosporins, like cephalexin and cefazolin, are most effective against Gram-positive bacteria. Later generations (up to the fifth generation) have increased activity against Gram-negative bacteria and improved resistance to beta-lactamase enzymes [10,25,26]. Examples of antibiotics from the cephalosporin group are shown in Figure 4. Toxicological data, including molecular formulas, CAS numbers, LD50 values, and PubChem references for the antibiotics shown, are summarized in Table 2 [27,28,29,30,31,32,33].

Monobactams contain a single antibiotic, Aztreonam, which is structurally different from other beta-lactams due to its monocyclic beta-lactam ring (Figure 2). Monobactams are primarily active against aerobic Gram-negative bacteria, including *Pseudomonas aeruginosa*, and are useful for patients with penicillin allergies [6].

## 4. Extraction Methods

Sample preparation plays a critical role in the analysis of antibiotics in various matrices. Matrices often contain compounds, such as protein, fat, sugar, and inorganic salts, that can hinder the detection of antibiotics, leading to inaccurate results. Impurities can influence the separation of target compounds, cause matrix effects, and lower the sensitivity of detection. To meet these challenges, several pretreatment methods are commonly used: liquid–liquid extraction (LLE), solid-phase extraction (SPE), ion-paired extraction (IPE), fast, easy, cheap, effective, robust, and safe (QuEChERS), matrix solid-phase dispersion (MSPD), dispersive-solid phase extraction (d-SPE), molecularly imprinted polymer extraction (MIP), magnetic molecularly imprinted polymer extraction (MMIP), and various microextraction methods [34]. The choice of technique depends on the matrix being tested, the properties of the antibiotics of interest, and the detection method used. Each of these techniques has its specific advantages and disadvantages.

Liquid–liquid extraction (LLE) is one of the earliest developed sample preparation techniques, which is still widely used, particularly in the analysis of biological samples. It involves transferring the analyte from one liquid phase, usually aqueous, to another liquid phase, typically an immiscible organic solvent, based on the differences in solubility of the substance between the two phases. However, this method has several drawbacks. It can lead to the formation of emulsions and requires large sample volumes and toxic organic solvents, which result in significant waste and environmental pollution. Additionally, LLE is costly, time-consuming, and unsuitable for hydrophilic compounds [35,36].

Common solvents used in extraction include acetonitrile, methanol, and ethyl acetate. Acetonitrile allows for high recovery rates while minimizing the co-extraction of matrix components, and it also denatures proteins and inactivates enzymes. In contrast, methanol and ethyl acetate tend to extract a greater amount of matrix components. Therefore, in multicomponent analyses, it is crucial to strike a balance between recovery and extract purity [37,38].

Over the years, various methods for extracting antibiotics using different solvents have been developed. Acetonitrile has been used, among other solvents, to detect different groups of antibiotics, including β-lactams [39,40]. However, in the case of β-lactams, the use of methanol may lead to their degradation, and acetonitrile is often not an efficient solvent for their extraction [41]. Rocco and colleagues [41] proposed an effective extraction method using a mixture of water, acetonitrile, and dimethyl sulfoxide (DMSO). LLE with trichloroacetic acid has also been used for the extraction of β-lactams and other antibiotics from fish samples [42].

In the ion-paired extraction (IPE) technique, a variant of liquid–liquid extraction (LLE), the extraction of ionic compounds is achieved by converting them into neutral forms. Positively charged ions react with appropriate organic anions, while negatively charged compounds interact with organic cations, leading to the formation of molecules that are soluble in nonpolar organic solvents. As a result, the modified compounds can be effectively extracted from aqueous solutions [43].

To enhance the efficiency of the ion-pair formation process, specific reagents are used in IPE, which facilitate both the creation of ion pairs and the stabilization of the resulting complexes in the organic phase. Examples of such reagents include heptafluorobutyric acid, bis(2-ethylhexyl)phosphate, dioctylsulphosuccinate sodium salt, trifluoroacetic acid, and pentafluoropropionic acid. Their use not only enables the formation of ion pairs but also promotes the effective transfer of analytes into the organic solvent, thereby improving the extraction efficiency [44,45].

The QuEChERS method (quick, easy, cheap, effective, rugged, and safe) is based on liquid–liquid extraction techniques and is a modern approach to sample preparation, widely used in the analysis of pesticide residues in food [2,46]. Due to its advantages in line with the principles of “green chemistry”, this method quickly found application in the extraction of other contaminants, such as antibiotics, from various matrices. QuEChERS offers an eco-friendly alternative to traditional methods, providing fast and energy-efficient solutions without compromising analytical performance [47].

The QuEChERS method consists of an extraction and purification step. In the first step, the sample is mixed with acetonitrile and salts (most commonly anhydrous MgSO_4_ and NaCl). In the next step, the organic phase is purified using dispersive solid-phase extraction (d-SPE). Sorbents such as PSA and C18 are typically used to effectively remove fatty acids and other impurities, while anhydrous MgSO_4_ is used to eliminate residual water from the extract [48,49,50]. Key parameters of the method, such as solvents, extraction conditions, or sorbent types, can be easily adjusted and optimized depending on the sample being tested, making it effective in detecting antibiotics in various animal-derived matrices [51]. One modification involved the use of acetonitrile with formic acid for the extraction of 16 β-lactams from chicken muscle, with purification carried out using C18, PSA, and anhydrous MgSO_4_ [52]. In another study, a mixture of acetonitrile and water was employed for the extraction of cephalosporins from beef, followed by purification using PSA, C18, and anhydrous MgSO_4_ [53]. To analyze 23 β-lactams in eggs, raw milk, formula milk, and various meat and fish products, including baby food, acetonitrile with the addition of Na_2_SO_4_ and NaCl was used, while purification was performed using Na_2_SO_4_ and C18 sorbent [54]. These examples demonstrated that the QuEChERS method can be easily modified to suit different types of antibiotics and sample matrices, making it a versatile approach for various analytical applications.

Matrix solid-phase dispersion (MSPD) is an alternative to the QuEChERS method and was popular in residue analysis during the 1980s and 1990s. This technique enables simultaneous extraction of the sample and isolation of analytes in a single step. MSPD procedures involve the use of dispersing sorbents with chemically modified silica surfaces, such as C18 or C8 [55]. In the MSPD method, sorbents with particle sizes ranging from 40 to 100 μm are most commonly used. The sample is mixed with the sorbent in a ratio typically ranging from 1:1 to 1:4 (usually 0.5 g of sample and 2 g of dispersing sorbent) using a mortar and pestle made of glass or agate. After mixing, the sample–sorbent mixture is air-dried for 5–15 min and then placed between two frits in a syringe barrel and compressed using a plunger. To eliminate the air-drying step, untreated silica or sodium sulfate (Na_2_SO_4_) have been introduced as dispersing agents [37,43]. In the MSPD method, nonpolar solvents, such as hexane, are used to remove lipophilic matrix interferences, while polar solvents, like dichloromethane, alcohols, or hot water, are applied for the extraction of veterinary drugs, with caution to avoid analyte degradation.

Direct contact between the entire surface of the sample and the solvent allows for more effective washing and elution, and the use of solvents with different polarities enables sequential elution of compounds. An additional advantage of MSPD is the elimination of the need for protein precipitation and centrifugation steps [43]. Solid-phase extraction (SPE) is a key technique in analytical chemistry, particularly useful for analyzing trace amounts of substances like pharmaceuticals and pesticides in various matrices [43]. SPE allows for the enrichment of trace amounts, sample purification, and medium exchange. This method stands out for its low cost, short analysis time, high selectivity due to the wide range of available solid sorbents, and its reduced solvent consumption. It is also easy to automate [56,57]. However, SPE techniques have some limitations, such as unstable sorptive properties, variability between batches of sorbents with chemically modified surfaces, and a higher risk of contamination (originating from production materials) [57]. The choice of sorbent in the SPE method is crucial, as it affects key parameters like selectivity and affinity. The effectiveness of SPE largely depends on matching the sorbent to the properties of the analyte and the matrix. The decision should be based on the physicochemical characteristics of the analytes and the matrix, which define the interactions between the analyte and the sorbent [37]. This method offers a wide range of sorbents, including reversed-phase sorbents, normal-phase sorbents, ion-exchange sorbents, mixed-mode sorbents, functionalized polymer resins, immunosorbents, and molecularly imprinted polymers (MIPs) [58].

SPE is widely used in the analysis of antibiotic residues in various matrices due to its flexibility in adjusting parameters, such as sorbent selection, eluent composition, and purification conditions. Oasis HLB columns are frequently cited in the literature for their ability to retain both polar and nonpolar compounds, making them effective for analyzing a wide range of antibiotics [38,59,60,61,62,63]. Additionally, newer Oasis PRiME HLB columns have been introduced, which do not require conditioning and washing prior to elution [64,65,66]. Strata-X columns, chemically modified polymeric sorbents, are also commonly used for the analysis of antibiotics in complex matrices, offering high selectivity and chemical resistance [67,68].

SPE methods, due to their versatility and ability to selectively extract compounds, have become an essential tool in the analysis of antibiotic residues in biological materials.

Dispersive-SPE (d-SPE) is a simplified clean-up method in which a pre-extracted sample is mixed with a sorbent. The sorbent binds unwanted matrix components, allowing the target analytes to remain in the solution. After brief centrifugation, the resulting supernatant can be directly analyzed or concentrated if needed. This technique is characterized by its speed, simplicity, and low cost. Although it does not provide as thorough clean-up as traditional SPE, its efficiency, reproducibility, and ease of use make it a highly practical solution [43].

Molecularly imprinted polymers (MIPs) are synthetic materials that selectively bind to specific molecules by precisely replicating their shape and functional groups. They are increasingly used for preparing samples from complex environmental matrices, offering temperature and pH stability, reusability, and shorter preparation times. MIP columns are also utilized for the analysis of antibiotics in various matrices [69,70,71].

Recent studies, including a recent review focused on the application of magnetic molecularly imprinted polymers (MMIPs) for the extraction of veterinary drug residues from milk, have described the development and application of MMIPs [72]. MMIPs are an advancement of traditional MIPs by incorporating magnetic particles, enabling easy separation using a magnetic field and further improving the sample preparation process [72].

Microextraction by packed sorbent (MEPS) is a miniaturized form of solid-phase extraction (SPE), in which a small amount of sorbent, approximately 2 mg, is placed directly inside the syringe needle [73]. Compared to conventional SPE techniques, MEPS allows for the use of significantly smaller sample and solvent volumes and reduces the sample preparation time from several minutes to just 1–2 min [74]. The technique enables simultaneous extraction, pre-concentration, and clean-up, with a very low dead volume (<10 μL) that facilitates automated sample handling [73]. MEPS is applicable for sample volumes ranging from 10 to 1000 μL and can be directly coupled with analytical techniques, such as liquid chromatography (LC), gas chromatography (GC), LC-MS, or GC-MS [37,73]. It is available with different sorbent types, including SCX, SAX, C18, C8, and silica, and can be used in reversed-phase, normal-phase, ion-exchange, and mixed-mode applications [74].

## 5. Extraction of β-Lactams

An overview of the extraction methodologies for the determination of β-lactam antibiotics in different matrices is presented in Table 3. The studies are discussed in chronological order.

Frédérique van Holthoon et al. [62] conducted a study in which eight kinds of penicillin (amoxicillin, ampicillin, penicillin G, penicillin V, oxacillin, cloxacillin, dicloxacillin, and nafcillin) were extracted from porcine muscle, kidney tissue, and milk. In this method, penicillins were converted to stable piperidine derivatives, followed by purification using solid-phase extraction with Oasis^®^ HLB cartridges. Analysis was carried out by LC-MS/MS using a Symmetry C18 column. Accuracy for the extraction procedure ranged from 94% to 113% (muscle), 83% to 111% (kidney), and 87% to 103% (milk).

Zhang et al. [69] developed a method to extract three β-lactam antibiotics (penicillin V, amoxicillin, and oxacillin) from bovine milk using magnetic molecularly imprinted polymer (MMIP) extraction. Analysis was carried out by LC-MS/MS using a Symmetry C18 column. The obtained relative standard deviations of intra- and inter-day fluctuated between 3.2% and 8.3%, as well as between 3.6% and 9.8%. The β-lactam antibiotic recoveries obtained using this method ranged from 71.6% to 90.7%.

Three β-lactam antibiotics were also successfully extracted, but this time from chicken muscles (ampicillin, amoxicillin, and penicillin G), along with other groups of veterinary drugs, using liquid–liquid extraction (LLE) with a mixture of 2% trichloroacetic acid aqueous solution and acetonitrile, and analyzed by LC-MS/MS using a ZIC-HILIC column. For the developed method, recoveries for β-lactam antibiotics in the examined tissues ranged between 64% and 87%, with precision ≤ 15% RSD, and the LOQ values were 8.5 µg/kg for amoxicillin, 5 µg/kg for ampicillin, and 5 µg/kg for penicillin G [75].

Studies were conducted to develop a method for the analysis of β-lactam antibiotics, where seven penicillins (ampicillin, dicloxacillin, penicillin G, amoxicillin, nafcillin, oxacillin, and cloxacillin) and seven cephalosporins (cephalexin, cefoperazone, cefazolin, cephapirin, ceftiofur, cefquinome, and cephalonium) were extracted from cow milk using solid-phase extraction with Oasis^®^ HLB cartridges and analyzed by LC-MS/MS using an Agilent Zorbax Eclipse XDB-C8 column and by UPLC-MS/MS using an Acquity UPLC BEH Shield RP18 column, with the presence of quinolones also being considered. The recoveries of β-lactam antibiotics were higher than 70% (except for amoxicillin), and for cephalosporins, the LOQ values for LC-MS/MS ranged from 0.3 to 125 µg/kg, while for penicillins, they ranged from 0.1 to 0.5 µg/kg. For UPLC-MS/MS, the LOQ values for cephalosporins ranged from 0.06 to 2.5 µg/kg, and for penicillins from 0.1 to 9 µg/kg [76].

Six β-lactam antibiotic residues (ceftiofur, penicillin G, penicillin V, oxacillin, cloxacillin, and dicloxacillin) were isolated from bovine milk using a rapid liquid–liquid extraction method with acetonitrile as the extraction solvent, developed by Jank et al. [77]. The compounds were analyzed by LC-MS/MS using a Synergy C_18_ column. The average accuracies obtained for the extracted compounds ranged from 104.1% to 109.9%, with LOQ values ranging from 1.0 ng/mL to 25 ng/mL.

Another research group, led by Pérez-Burgos [53], developed two methods for the extraction of cephalosporins from beef muscle (cephalexin, cephalonium, cefoperazone, cephapirin, cefquinome, cephazolin, and ceftiofur). The extraction and LC-MS/MS analysis were the same for both methods, with the difference being in the sample clean-up process. In the first approach, the QuEChERS method was applied using dispersive SPE kits containing PSA, C18 sorbents, and MgSO_4_. In the second approach, SPE was performed using Isolute ENV+ cartridges. Chromatographic separation was carried out on a Zorbax Eclipse XDB-C8 column with a Kromasil C8 precolumn. Comparing accuracies, the QuEChERS method provided slightly better results than the SPE method, with recovery only lower than 80% for cephalexin. The LOQ for the SPE method ranged from 0.1 to 10 µg/kg, while for the QuEChERS method, it ranged from 1.5 to 50 µg/kg, with the highest values obtained for cephalexin.

Muscle tissues from cattle, pigs, and chickens were utilized for studies on the extraction method of nine penicillin antibiotics, including penicillin G, penicillin V, amoxicillin, ampicillin, nafcillin, oxacillin, cloxacillin, dicloxacillin, and piperacillin (used as an internal standard). Extraction was carried out using LLE with water and acetonitrile, followed by sample purification using SPE with Isolute ENV+ cartridges. Chromatographic separation was performed on a Zorbax Eclipse XDB-C8 column with a Kromasil C8 precolumn. For the developed method, recoveries for all antibiotics in the examined animal tissues were obtained above 70%, except for amoxicillin at 50% recovery. The LOQ values ranged from 0.2 to 8 µg/kg, with the highest values observed for ampicillin and penicillin G in pig tissues [78].

Penicillin G, oxacillin, and cloxacillin were extracted from beef and milk using ion-pair extraction and a binary water–acetonitrile mixture. The first step was the removal of proteins and fats using acetone–acetonitrile solutions. Chromatographic separation was performed on an Xbridge™ C18 reversed-phase column, and detection was carried out by HPLC-UV at 215 nm. This method achieved detection limits (LOD) at the level of 1–2 ng/mL, good repeatability with RSD below 2%, and the average recoveries were higher than 85%. The LOQ values were 3 ng/mL for penicillin G, 3 ng/mL for oxacillin, and 7 ng/mL for cloxacillin [79,80].

Using an ultrasound-assisted matrix solid-phase dispersive extraction method, it was possible to successfully extract four penicillins, including amoxicillin, oxacillin, cloxacillin, and dicloxacillin, along with eight cephalosporins, such as ceftiofur, cefuroxime, cefotaxime, cefoperazone, cefaclor, cefadroxil, cephalexin, and cefazolin, from milk. The matrix solid-phase dispersive extraction (MSPD) procedure was conducted using the Oasis HLB sorbent. Chromatographic separation was performed by HPLC-PDA on an Inertsil ODS-3 column. Using this method, the recoveries for the analyzed antibiotics ranged from 85.0% to 115.7%, and the values of the relative standard deviation (RSD) were less than 12.7%. The LOQ values ranged from 19.2 to 46.5 µg/kg [81].

Maggi et al. [82] conducted research on catfish to develop an extraction method for eight penicillins (amoxicillin, ampicillin, cloxacillin, dicloxacillin, nafcillin, oxacillin, penicillin G, and penicillin V) using an online solid-phase extraction method with a C18 cartridge, followed by analysis with liquid chromatography coupled to ion trap tandem mass spectrometry. The symbiosis system of online SPE contained an automatic device that loaded, cleansed, and eluted the SPE cartridge and transferred analytes directly onto the column for analysis. Chromatographic separation was carried out on a Synergy Max column. The recoveries of β-lactam antibiotics at spike levels of 2.0, 10.0, and 50 µg/kg^−1^ varied between 72% and 92%. The samples showed precision values lower than 20%, except for amoxicillin.

In a targeted analysis of β-lactam antibiotic residues, twenty-two compounds were extracted from poultry muscle, including penicillins (amoxicillin, ampicillin, penicillin G, penicillin V, cloxacillin, dicloxacillin, nafcillin, and oxacillin), cephalosporins (ceftiofur, cefquinome, cefapirin, cefalexin, cefalonium, cefazolin, cefacetrile, and cefoperazone), and carbapenems (biapenem, doripenem, ertapenem, imipenem, meropenem, and faropenem). The sample purification was performed using solid-phase extraction (SPE) with a Phenomenex Strata-X cartridge. Analysis was carried out by a LC-MS/MS system. Separation was carried out using a Waters Acquity UPLC CSH C18 analytical column. The technique demonstrated acceptable quantitative results for all substances, achieving trueness ranging from 80% to 110% and within-laboratory reproducibility under 22% at the target concentration, with the exception of biapenem. For biapenem, the method was found to be appropriate only for qualitative analysis [68].

Nine β-lactam antibiotics (ampicillin, penicillin G, penicillin V, cephalexin, cefazolin, cefoperazone, cloxacillin, dicloxacillin, and oxacillin) were successfully extracted from ewe milk, as demonstrated by Cámara et al. [83]. The extraction was performed using SPE with Spe-ed C18 cartridges. Chromatographic analysis was carried out using an LC-UV-DAD system on a Supelcosil LC 18 DB column. The recovery of the studied β-lactams ranged from 79% to 96% with standard deviations between 0.5% and 4.9%. The LOQ values ranged from 3.4 to 8.6 μg/kg.

A research group led by Jank [40] successfully extracted fourteen β-lactam antibiotics (ampicillin, amoxicillin, penicillin G, penicillin V, oxacillin, cloxacillin, dicloxacillin, cefalonium, cefoperazone, cefapirin, ceftiofur, cefquinome, cefalexin, and nafcillin) from bovine milk using LLE. After extraction, the analytes were purified by adding C18 bulk sorbent directly to the sample. The analysis by LC-MS/MS was operated in positive mode. Chromatographic separation was obtained using a DuraShell RP column paired with a SecurityGuard Cartridge C18 guard column. Accuracy values ranged between 92% and 110%.

Dorival-García et al. [84] focused on extracting fourteen β-lactam antibiotics (cefoperazone, cephalexin, ceftiofur, cefazolin, cephapirin, cefquinome, cephalonium, amoxicillin, nafcillin, oxacillin, cloxacillin, ampicillin, dicloxacillin, and penicillin G) from raw cow milk. The extraction was performed using ultrasound-assisted extraction combined with dispersive-SPE, involving McIlvaine buffer (pH 6.0) and an acetonitrile:methanol mixture, followed by clean-up with PSA and anhydrous MgSO_4_. The analysis was performed by UHPLC-MS/MS with chromatographic separation achieved on an Acquity UPLC BEH C18 column. The recovery of the studied β-lactams ranged from 96.0% to 104.5%, with LOQ values ranging from 0.3 to 1.3 ng/g.

Using molecularly imprinted polymer (MIP) extraction, six β-lactam antibiotics (cephalexin, ceftiofur, cefazolin, cephapirin, cefquinome, and cephalonium) were extracted from bovine milk. The extraction was followed by analysis using a UHPLC-MS/MS system. Separation was achieved using an Acquity UPLC BEH™ C18 column. The results of the repeatability study showed that mean recoveries ranged from 62% to 100%, with RSDs below 6% for cephapirin, cephaloniu, cefquinome, and cefthiofur. Significantly lower values were obtained for cephalexin and cefazolin (recoveries: 15–28%), with RSD values lower than 6%. The LOQ for the analyzed compounds ranged from 0.4 to 12.5 µg/kg [70].

Another example of β-lactam extraction is a method that isolates sixteen β-lactam antibiotics, including penicillin G, cefalexin, ampicillin, penicillin V, penenethicillin, amoxicillin, methicillin, oxacillin, naficillin, cephapirin, cloxacillin, cefazolin, azlocillin, dicloxacillin, piperacillin, and ceftiofur, from pork muscle. The extraction was performed using matrix solid-phase dispersion with Oasis HLB sorbent, which was previously conditioned with acetonitrile, water, and sodium chloride solution. The analysis was carried out using a UPLC-MS/MS system, and chromatographic separation was performed on an Acquity UPLC HSS T3 C18 column (1.7 µm, 2.1 × 100 mm). The recoveries of β-lactam antibiotics were between 92% and 111%, and the RSDs were lower than 12%, with LOQ values ranging from 0.07 to 2.10 µg/kg [85].

Moretti et al. [86] successfully extracted 17 β-lactam antibiotics (desacetylcephapirin, amoxicillin, cephaphirin, cefquinome, cefacetrile, cefalonium, cefalexin, cefazolin, ampicillin, cefoperazone, ceftiofur, penicillin G, oxacillin, penicillin V, cloxacillin, dicloxacillin, and nafcillin) from meat using LC-MS/MS analysis, as part of a multiclass method detecting 62 antibiotics. The extraction involved the addition of EDTA, followed by LLE using an acetonitrile–water mixture (80:20, *v*/*v*) and pure acetonitrile. The analytes were separated using the Poroshell 120 EC-C18 column (100 × 3.0 mm; 2.7 μm) and Poroshell (2.1 × 5 mm) guard column. The average recoveries for β-lactam antibiotics in meat using this method ranged between 59% and 87%, with LOQ values of 2 µg/kg for all compounds, except for cefacetrile, which had an LOQ of 10 µg/kg.

Rocco et al. [41] used UHPLC-MS/MS detection to extract thirty β-lactam antibiotics, including cefadroxil, cefazolin, cephalexin, cefacetrile, cefalonium, cefoperazone, cefotaxime, cefquinome, cefuroxime, desacetyl cephapirin, desfuroylceftiofur cysteine disulfide, desfuroylceftiofur dimer, biapenem, doripenem, ertapenem, imipenem, meropenem, faropenem, amoxicillin, ampicillin, cloxacillin, dicloxacillin, mecillinam, methicillin, nafcillin, oxacillin, penicillin G, penicillin V, piperacillin, and ticarcillin, from bovine muscles. The extraction was performed by liquid–liquid extraction using water and acetonitrile, followed by a purification step with C18 sorbent. Separation was achieved with a stainless-steel CSH C_18_ column. The technique demonstrated trueness, which ranged between 69% and 143%. Precision was between 2.0% and 29.9% (within-laboratory reproducibility conditions).

In an effort to develop a method for detecting antibiotic residues in poultry, sixteen β-lactam antibiotics (ampicillin, amoxicillin, azlocillin, cefalexin, cefazolin, ceftiofur, cephapirin, cloxacillin, dicloxacillin, methicillin, nafcillin, oxacillin, penicillin G, penicillin V, penenethicillin, and piperacillin) were extracted from chicken muscle using UPLC-quadrupole-Orbitrap-MS. The QuEChERS method included extraction with acetonitrile containing 0.1% formic acid, using anhydrous magnesium sulfate and sodium acetate, followed by clean-up with end-capped C18, PSA, and anhydrous magnesium sulfate sorbents. Separation was accomplished using an HSS T3 C18 column. The recoveries ranged between 83% and 112% and the RSDs were < 15%, and LOQ values ranged from 0.03 to 0.59 µg/kg [52].

Turnipseed et al. [64] analyzed various fish species, including salmon, tilapia, and catfish, as well as shrimp and eel, to extract, among others, eight β-lactam antibiotics (amoxicillin, ampicillin, aspoxicillin, cloxacillin, dicloxacillin, oxacillin, penicillin G, and penicillin acid), using a multiclass method that also detects other veterinary drugs, using extraction with acetonitrile containing 0.2% p-toluenesulfonic acid monohydrate and 2% glacial acetic acid, followed by clean-up on an Oasis PRiME HLB cartridge. LC separation was carried out with the Supelco Ascentis Express C18 column, and the analysis was performed using LC-Q-Orbitrap HRMS. The average recoveries of the studied β-lactams were >90%, with standard deviations < 15%.

Seven β-lactam antibiotics (amoxicillin, ampicillin, cloxacillin, dicloxacillin, benzylpenicillin, cefquinome, and cefalexin) were extracted from heavy pigs’ urine and muscle using HPLC-MS/MS analysis. There were two methods of sample extraction, a different one for each matrix. For urine samples, SPE was applied after direct sample loading onto Oasis HLB cartridges, while for muscle samples, additional deproteinization and defatting steps were performed before SPE. Analytical separation was conducted using a Synergi Hydro-RP column and a Phonomenex C18 guard column. The mean recoveries for β-lactam antibiotics in the analyzed matrices ranged between 90% and 107% [61].

Bessaire et al. [54] extracted twenty-three β-lactam antibiotics (amoxicillin, ampicillin, aspoxicillin, cefacetril, cefadroxil, cefalexin, cefalonium, cefapirin, cefazolin, cefoperazone, cefquinome, ceftiofur, cefuroxime, cloxacillin, desacetylcefapirin, dicloxacillin, nafcillin, oxacillin, penicillin G, penicillin V, piperacillin, sulbactam, and tazobactam) from various foods of animal origin, including eggs, raw milk, processed dairy ingredients, infant formula, and meat- and fish-based products. The QuEChERS method involved extraction with acetonitrile in the presence of Na_2_SO_4_ and NaCl, followed by clean-up with Na_2_SO_4_ and C18 sorbents. Analysis was performed by LC-MS/MS using an Acquity BEH VanGuard precolumn attached to an Acquity BEH C18 column. Using this method, the average recoveries for the analyzed antibiotics ranged between 34% and 94%, with an average CV of 11%.

LC-MS/MS was used to detect five β-lactam antibiotics (ampicillin, cefazolin, oxacillin, penicillin G, and penicillin V), which were successfully extracted from fish, along with other groups of antibiotics. The extraction process for the antibiotics in the samples was carried out according to the previously developed method described by Gaugain-Juhel et al. Liquid–liquid extraction was performed using 5% trichloroacetic acid (TCA). A Millipore PVDF membrane with 0.45 μm pore size was used to filter the extract before LC-MS/MS analysis [39,42]. The separation was performed using an Agilent Technologies Zorbax Eclipse XDB C18. Even though the sensitivities of these analytes at a concentration of 0.5 MRL were satisfactory (greater than 95%), most of them exhibited high limit of detection (LOD) values, occasionally exceeding the MRL. Therefore, although the method is capable of monitoring these compounds, it cannot detect them at concentrations below the MRL [42].

A research group led by Karageorgou [87] extracted eight β-lactam antibiotics (amoxicillin, ampicillin, oxacillin, cloxacillin, ceftiofur, cefalonium, cefazolin, and cefapirin) from raw milk using the HPLC-DAD method. The extraction and purification were carried out using dispersive solid-phase extraction with preconditioned Plexa sorbent combined with QuEChERS salts containing magnesium sulfate, PSA, and C_18_EC. Separation was carried out using a Perfectsil ODS-2 analytical column. Counting of target analytes was conducted at the wavelength of optimum absorbance for each analyte: amoxicillin, ampicillin, cloxacillin, and oxacillin (240 nm), cefazolin, cefapirin, ceftiofur, and cefalonium (265 nm). The mean recovery rates ranged from 81.8% to 116.9%, with RSDs below 10.7% for all the examined agents.

A method for the extraction of eleven β-lactam antibiotics (amoxicillin, ampicillin, cefazolin, cephapirin, desacetyl cephapirin, cloxacillin, ceftiofur metabolite, dicloxacillin, nafcillin, oxacillin, and penicillin G), along with other classes of drugs, was developed for use with bovine tissues, including kidney, liver, and muscle. Liquid–liquid extraction was performed using a mixture of acetonitrile and water. The analysis was carried out by LC-MS/MS, and chromatographic separation was performed using a Waters Acquity HSS T3 analytical column and a 0.5 cm Waters Vanguard column. The average recoveries for β-lactam antibiotics ranged between 70% and 120% for most of the analytes, except for a few instances where the recoveries were higher or lower than the given range. The LOQ values ranged from 0.7 to 14 ng/g for kidney, from 1 to 22 ng/g for liver, and from 1 to 33 ng/g for muscle, with the highest values consistently observed for amoxicillin [88].

To extract seventeen β-lactam antibiotics, such as desacetylcephapyrin, amoxicillin, cephapyrin, cefquinome, cefacetrile, cefalonium, cefalexin, cefazolin, ampicillin, cefoperazone, ceftiofur, penicillin G, oxacillin, penicillin V, cloxacillin, dicloxacillin, and nafcillin, along with other classes of antibiotics, from muscle and milk, 1.50 g of each sample was weighed and placed into a Falcon tube. The sample preparation was first described by Moretti et al. [86,89,90]. The extraction involved LLE with acetonitrile/water and EDTA for muscle samples, and with acetonitrile and EDTA for milk samples, followed by a second extraction with acetonitrile for both matrices. LC-MS/MS detection was used. Separation was performed on an Agilent Poroshell 120 EC-C18 column with an Agilent Poroshell guard column. Using this method, the recoveries for the analyzed antibiotics ranged from 61% to 110%. The lowest recovery (61%) applied to ceftiofur extracted from milk, whereas the highest recovery (110%) applied to cloxacillin extracted from milk [89].

Studies have also been conducted on the extraction from chicken feathers. A research group led by Gajda [63] successfully extracted fifteen β-lactam antibiotics—amoxicillin, ampicillin, penicillin G, penicillin V, oxacillin, cloxacillin, nafcillin, dicloxacillin, cephapirin, cefoperazone, cephalexin, cefquinome, cefazolin, cefalonium, and ceftiofur, along with other classes of antibiotics. The extraction was performed using LLE with acetonitrile in the presence of oxalic acid and Na_2_EDTA, followed by SPE using Oasis HLB cartridges. The analysis was performed by UHPLC-MS/MS using an Agilent Zorbax SB-C18 and a Phenomenex octadecyl guard column for the separation. The gradient mobile phase consisted of acetonitrile and 0.025% HFBA. The recoveries for β-lactams in analyzed tissues ranged between 95% and 108%.

Di Rocco et al. [91] conducted a study to extract 32 β-lactam antibiotic residues from milk, including 12 penicillins (amoxicillin, ampicillin, cloxacillin, dicloxacillin, mecillinam, methicillin, nafcillin, oxacillin, penicillin G, penicillin V, piperacillin, and ticarcillin), 14 cephalosporins (cefacetrile, cefadroxil, cephalexin, cephapirin, cefalonium, cefazolin, cefoperazone, cefotaxime, cefquinome, ceftiofur, cefuroxime, desacetyl cephapirin, desfuroylceftiofur cysteine disulfide, and desfuroylceftiofur dimer), 5 carbapenems (biapenem, doripenem, ertapenem, imipenem, and meropenem), and faropenem. The extraction was performed by LLE (with acetonitrile) and purification by d-SPE (C18 sorbent). UHPLC-MS/MS detection was applied. The separation was carried out using an Agilent Zorbax Eclipse Plus Phenyl-Hexyl Rapid Resolution HD analytical column. Under conditions of within-laboratory repeatability, the accuracy ranged from 91% to 130%, while the precision varied from 1.4% to 38.6%.

Various animal-derived food products, including meat, kidneys, liver, bacon, milk, eggs, and honey, were used to extract residues of nineteen β-lactam antibiotics: amoxicillin, ampicillin, dicloxacillin, carbenicillin, cloxacillin, nafcillin, oxacillin, penicillin G, penicillin V, piperacillin, ticarcillin, cephalexin, cefalonium, cefoperazone, cefapirin, cefquinome, cefotaxime, ceftiofur, and cefuroxime. These residues were then analyzed using UHPLC-Q-TOF. Two extraction procedures based on LLE were applied, one using acetonitrile in the presence of NaCl and EDTA (first option), and the other using water with succinic acid and EDTA, followed by acetonitrile and ammonium sulfate (second option). The separation was performed with Acquity UPLC^®^ BEN C18 columns. The second option of sample preparation was more suitable for milk, meat, liver, kidneys, eggs, and bacon, whereas the first option was preferable for honey (a larger number of analytes exhibited a matrix effect of less than 80%). Using this method, the recoveries for the analyzed antibiotics from different matrices ranged from 75% to 110%, and the values of RSD were ≤ 11% under the conditions of matrix calibration [92].

Cheng et al. [71] extracted ceftiofur from milk and muscle samples of chicken, pork, and beef using MISPE (molecularly imprinted solid-phase extraction), followed by HPLC-UV detection. For this purpose, after sample preparation, the analytes were loaded onto a cartridge packed with imprinted polymer, conditioned with methanol and water. The collected eluents were analyzed using HPLC-UV. Separation was performed using a reversed-phase C18 cartridge. UV detection was carried out at 292 nm. The average recoveries obtained using this method were greater than 91.9%, and the RSD was less than 8.5%, with an LOQ of 0.005 mg/L.

Sixty antibiotics, including sixteen β-lactam antibiotics (desacetylcephapirin, amoxicillin, cephapirin, cefquinome, cefalonium, cefalexin, cefazolin, ampicillin, cefoperazone, ceftiofur, penicillin G, oxacillin, penicillin V, cloxacillin, dicloxacillin, and nafcillin), were extracted from eggs. Two extraction steps based on LLE were applied: first using a mixture of acetonitrile and water with 0.05% formic acid in the presence of Na_2_EDTA, and second with pure acetonitrile. UHPLC-HRMS detection was applied. The separation was performed using a Poroshell 120 EC-C18 column and an Agilent Technologies Poroshell guard column. The recoveries for the antibiotics in the analyzed tissues ranged from 65% to 91% [93].

Hu et al. [65] extracted four β-lactam antibiotics (cloxacillin, dicloxacillin, penicillin G, and penicillin V), along with other classes of antibiotics, from cereals, meat, eggs, milk, vegetables, and fruits. Three extraction steps based on LLE were performed: the first with acetonitrile:water containing 0.1% formic acid, the second with acetonitrile:water containing 0.2% formic acid and a DisQue salt pack (MgSO_4_ and sodium acetate), and the third with acetonitrile:water containing 0.2% formic acid. Purification was carried out by SPE using Oasis PRiME HLB cartridges. UHPLC-MS/MS analysis was carried out. The separation was performed using the Waters BEH C8 column. The average recoveries at three spiking levels were as follows: 82 ± 26% for cereals, 77 ± 26% for meat, 70 ± 34% for eggs, 69 ± 31% for milk, 73 ± 29% for vegetables, and 62 ± 37% for fruits.

To detect fourteen β-lactam antibiotics, including amoxicillin, penicillin V, penicillin G, cloxacillin, oxacillin, dicloxacillin, ampicillin, nafcillin, cefazolin, cephalexin, ceftiofur, cefquinome, cefoperazone, and cefapirin, milk samples from cows, sheep, and goats were analyzed using an LC-Orbitrap-HRMS system, which, as a multiresidue method, also allowed for the detection of other antibiotics. The extraction was based on LLE with acetonitrile containing 2% formic acid and EDTA, followed by SPE clean-up on Oasis HLB PRiME cartridges. The separation was performed using a Poroshell 120 EC-C18 column with a Poroshell guard column. The method showed effectiveness at the target screening concentration, and the false positive rate was less than 5% [66].

Lakew et al. [94] extracted amoxicillin, ampicillin, and penicillin G (three β-lactam antibiotics, among other groups) from chicken tissues. The extraction was based on LLE using a mixture of acetonitrile and methanol. The analysis was performed using LC-UV detection. The separation was performed using Phenomenex Hypersil BDS-C18 in reversed-phase mode with isocratic elution. UV absorption was measured at a wavelength of 230 nm. The recoveries were acceptable, ranging from 96% to 102%. LOQs ranged from 0.297 to 0.574 μg/kg.

A research group led by Wu [95] used a modified QuEChERS extraction method to isolate a total of 52 β-lactam antibiotics, including cefetamet, cefotaxime, cefterampivoxil, faropenem, cefmenoxime, cefuroxime, cefazolin, cefathiamidine, cefodizime, ticarcillin, cefpiramide, cefamandole sodium, carbenicillin, cefoperazone, ceftiofur, penicillin g, methicillin, cefuroxime axetil, azlocillin, cefpodoxime proxetil, piperacillin, mezlocillin, cefetamet pivoxil, dicloxacillin sodium, flucloxacillin, cloxacillin, oxacillin sodium, penicillin v, cefadroxil, amoxicillin, ampicillin, cephradine, cephalexin, aspoxicillin, mecillinam, cefotiam, ceftizoxime, cefdinir, cefozopran, cilastatin, cefepime, ceftazidime, cefixime, ceftibuten, cefpirome, ceftriaxone, cefprozil hydrate, meropenem, aspoxicillin, nafcillin, biapenem, and cefapirin sodium, from samples of meat and poultry, aquatic products, milk, and eggs. The extraction was carried out by a modified QuEChERS method, involving extraction with a mixture of acetonitrile and water and clean-up with octadecyl silica (C18) sorbent. The filtered extract was injected into the UPLC-MS/MS system. Separation was carried out using an Agilent Zorbax SB-Aq column. The mean recoveries of β-lactam antibiotics at spike levels of 10, 20, and 50 µg/kg varied between 67.1% and 109.8%, with relative standard deviations ranging from 0.5% to 9.9%. LOQs of β-lactams ranged from 0.07 to 0.97 mg/kg.

The methods described in the paper, such as LC-MS/MS and extraction techniques, including LLE, SPE, and QuEChERS, have a great potential to become standard procedures in routine laboratory testing. With their high sensitivity, specificity, and ability to detect low concentrations of β-lactam antibiotics and other antibiotic groups in various matrices, they provide reliable results. This is important for meeting legal requirements concerning antibiotic residues in animal-derived food.

Routine laboratories can successfully implement these techniques, as equipment like HPLC and LC-MS/MS are widely available, and the extraction methods are relatively easy to apply. Additionally, they feature short analysis times and good repeatability of results, which makes them efficient tools for everyday laboratory work.

## 6. Conclusions

In conclusion, recent advancements in extraction and detection techniques for β-lactam antibiotics have significantly improved the sensitivity, precision, and reliability of analytical methods. Despite these achievements, the complexity of food matrices continues to pose significant challenges, often leading to unpredictable matrix effects that can interfere with accurate quantification. Therefore, further research is necessary to refine existing methods and develop more robust solutions to overcome these limitations.

Techniques such as LLE, SPE, IPE, QuEChERS, MIP, and MSPD are continuously being improved to optimize recovery rates and reduce matrix effects. LC-MS/MS, with its high sensitivity and selectivity, has become a cornerstone in the analysis of antibiotic residues, enabling increasingly lower detection limits and high precision and becoming essential for ensuring food safety as well as regulatory compliance.

As the field progresses, it is essential to further improve extraction techniques and enhance the performance and sensitivity of LC-MS/MS equipment to address the challenges posed by increasingly complex food matrices and emerging contaminants. Ongoing innovations underscore the urgent need for research focused on harmonizing and standardizing analytical methods across laboratories, as well as developing more selective and efficient extraction procedures. These advancements would enable the detection of progressively lower concentrations of substances, the identification of new compounds, and the development of multiresidue methods that facilitate the simultaneous analysis of multiple substance groups. Progress in these areas will directly impact the effectiveness of food safety monitoring and support regulatory decision-making, thereby contributing to enhanced public health protection.

Furthermore, collaboration between international research institutions is essential to facilitate the standardization of methods and ensure global applicability of food safety monitoring systems. Wider adoption of these improved extraction techniques and analytical methods will not only enhance food safety but also contribute to more equitable access to safe food products globally.

## Figures and Tables

**Table 1:** Data for penicillins, including molecular formulas, CAS numbers, LD50 values (oral, rat), and PubChem references.

Antibiotic	Molecular Formula	CAS Number	LD50 (mg/kg) (Oral, Rat)	PubChem CID
**Penicillin G**	C_16_H_18_N_2_O_4_S	61-33-6	8000	5904 [15]
**Penicillin V**	C_16_H_18_N_2_O_5_S	87-08-1	>2220	6869 [16]
**Amoxicillin**	C_16_H_19_N_3_O_5_S	26787-78-0	>15,000	33613 [17]
**Ampicillin**	C_16_H_19_N_3_O_4_S	69-53-4	>5000	6249
**Oxacillin**	C_19_H_19_N_3_O_5_S	66-79-5	10,000	6196 [19]
**Cloxacillin**	C_19_H_18_ClN_3_O_5_S	61-72-3	n.a.	6098 [20]
**Nafcillin**	C_21_H_22_N_2_O_5_S	147-52-4	n.a.	8982 [21]
**Dicloxacillin**	C_19_H_17_Cl_2_N_3_O_5_S	3116-76-5	n.a.	18381 [22]
**Piperacillin**	C_23_H_27_N_5_O_7_S	61477-96-1	n.a.	43672 [23]
**Carbenicillin**	C_17_H_18_N_2_O_6_S	4697-36-3	n.a.	20824 [24]

n.a.—Data for oral administration in rats not available; for cloxacillin, the LD_50_ value for intracerebral administration in mice is 8.1 mg/kg; for piperacillin, the LD_50_ value for intravenous administration in mice is 5000 mg/kg; for carbenicillin, the LD_50_ value for intravenous administration in mice is 2363 mg/kg.

**Table 2:** Data for cephalosporins, including molecular formulas, CAS numbers, LD50 values (rat), and PubChem references.

Antibiotic	Molecular Formula	CAS Number	LD50 (mg/kg) (Rat)	PubChem CID
**Cefapirin**	C_17_H_17_N_3_O_6_S_2_	21593-23-7	16,356 (oral)	30699 [27]
**Ceftiofur**	C_19_H_17_N_5_O_7_S_3_	80370-57-6	1250 (intramuscular)	6328657 [28]
**Cefquinome**	C_23_H_24_N_6_O_5_S_2_	84957-30-2	n.a.	5464355 [29]
**Cephalonium**	C_20_H_18_N_4_O_5_S_2_	5575-21-3	2750 (intraperitoneal)	21743 [30]
**Cefazolin**	C_14_H_14_N_8_O_4_S_3_	25953-19-9	n.a.	33255 [31]
**Cephalexin**	C_16_H_17_N_3_O_4_S	15686-71-2	>20,000 (oral)	27447 [32]
**Cefoperazone**	C_25_H_27_N_9_O_8_S_2_	62893-19-0	n.a.	44187 [33]

n.a.—Data for oral administration in rats not available; for cefazolin, LD_50_ values were reported for intravenous (3000 mg/kg) and intramuscular (4000 mg/kg) administration in mice.

**Table 3:** Extraction methodologies for the determination of β-lactams in different food samples.

Amount of Compounds	Matrix	Sample Preparation	Analytical Technique		Reference
			Equipment	Separation	
8 β-lactam antibiotics	porcine muscle and kidney tissue milk	Samples (2 g) were homogenized with phosphate buffer (2 mL, 200 mM, pH = 6.0). Internal standard mixture was added, incubated (30 min, room temperature), phosphate buffer (100 mM, pH = 8.0, 40 mL) was added, vortexed (30 s), piperidine (300 µL) was added, mixed (5 min), phosphoric acid (100 µL) was added, vortexed, heated in a water bath (85 °C, 5 min), and cooled. The samples were centrifuged (2700× *g*, 10 min). Supernatants were filtered through a cotton wool into tubes, purified (Oasis^®^ HLB 60 mg, 3 cm^3^), and conditioned (2 mL methanol, 2 mL water and 2 mL phosphate buffer 100 mM, pH = 8.0). The samples were loaded (10 mL) and subsequently washed (2 × 2 mL of water), dried, and eluted (3 mL methanol/water; 80/20, *v*/*v*). Then, evaporated (dryness, 60 °C in a stream of nitrogen) and redissolved (500 μL 2% acetonitrile). The porcine tissue method was applied with a few modifications. Internal standards were added to milk samples (4 g). Phosphate buffer (100 mM, pH = 8.0, 40 mL) was added, vortexed (30 s), piperidine (300 µL) was added, mixed (5 min), and phosphoric acid (100 µL) was added and vortexed. The samples were centrifuged (2700× *g*, 10 min). Supernatants were filtered through a cotton wool into tubes. After conditioning the SPE column, extract was applied (20 mL). Final residue was redissolved in 2% methanol (200 µL).	LC-MS/MS (ESI−)	Column Waters Symmetry C_18_ column (3.0 × 150 mm; 5 µm) Mobile phase Gradient: A: 0.2% formic acid in water B: 0.2% formic acid in acetonitrile/water (9/1, *v*/*v*)	[62]
3 β-lactam antibiotics	bovine milk	MMIPs (100 mg) were placed into a conical flask and conditioned (3.0 mL methanol, 3.0 mL water). Supernatant was isolated and separated and removed. Milk sample (2.0 mL) and hydrochloric acid aqueous solution (18.0 mL) at pH 5 were added. Ultrasound for 5 min. β-Lactam antibiotics were isolated. Supernatant solution was discarded. The MMIPs were washed with water (3.0 mL). The antibiotics were eluted from the MMIPs (3 × 1.0 mL methanol solution with 5.0% acetic acid). Ultrasound for 30 s during each elution step. The eluate merged and evaporated (nitrogen gas at 40 °C). The residue was reconstituted (1.0 mL of 0.1% formic acid methanol solution). The eluate was filtered (0.45 μm) and injected into the LC-MS/MS system for analysis.	LC-MS/MS (ESI+)	Column Waters Symmetry C_18_ column (150 mm × 4.6 mm, 5 µm) Mobile Phase Isocratic: 0.1% formic acid in water: methanol (40:60, *v*/*v*)	[69]
3 β-lactam antibiotics	Chicken muscles	Sample (5 g) was weighed into a centrifuge tube, and extraction solution (10 mL; 2% trichloroacetic acid aqueous solution mixed with acetonitrile in a 1:1 volume ratio) was added and vortexed for 30 s. The sample was mechanically shaken (10 min) and centrifuged (3400 rpm, 5 min). Supernatant was transferred to a centrifuge tube. Hexane (5 mL) was added. The mixture was vortexed (1 min) and centrifuged (2400 rpm, 5 min). The hexane layer was discarded. The sample (200 μL) was transferred to a centrifuge tube and diluted with 10% formic acid (800 μL) in water–acetonitrile (1:9, *v*/*v*). The extract was filtered (0.2 μm nylon syringe filter) for LC separation.	LC-MS/MS ESI+	Column SeQuant ZIC-HILIC column (2.1 mm × 100 mm; 3.5 µm) Mobile phase Gradient: A: 50 mM ammonium formate in water (pH 2.5) B: acetonitrile	[75]
14 β-lactam antibiotics	cow milk	Sample (2 g) was weighed. Phosphate solution 0.2 M at pH 10 (0.5 mL) was added. Sample was centrifuged, and the SPE process was performed (HLB cartridges). Samples were activated with methanol (1 mL), water (1 mL), and 0.1 M phosphate solution at pH 10 (1 mL). Cartridge was washed with water (3 mL). The analytes were eluted with methanol (2 mL). Extract was injected into the LC-MS/MS system.	LC-MS/MS (ESI+) UPLC-MS/MS (ESI+)	Column (LC-MS/MS) Agilent Zorbax Eclipse XDB-C8 column (5 µm, 4.6 × 150 mm) Akady Kromasil C8 precolumn (4.6 × 15 mm; 5 µm) Column (UPLC-MS/MS) Waters Acquity UPLC BEH Shield RP 18 column (1.7 µm, 2.1 mm × 50 mm) Mobile phase Gradient: A: 0.1% formic acid in water B: 0.1% formic acid in acetonitrile	[76]
6 β-lactam antibiotics	milk	Milk sample (2 mL) was extracted with 4 mL of acetonitrile. Acetonitrile (1 mL) was added to the sample and vortexed (10 s). This step was repeated three more times. Then, it was mixed (20 min), sodium chloride (1.0 g) was added, mixed (20 min), and centrifuged (5 min, 5000× *g*, 5 °C). 1 mL of the supernatant was collected for LC-MS/MS analysis.	LC-MS/MS (ESI+)	Column Phenomenex Synergy C_18_ column (3.0 × 150 mm; 4 µm) Phenomenex security guard system C_18_ (3.0 × 4.0 mm; 5 µm) Mobile phase Gradient: A: 0.1% formic acid in water B: 0.1% formic acid in acetonitrile	[77]
7 β-lactam antibiotics	beef muscle	Muscle sample (4 g) was homogenized. Internal standard mixture was added, kept in the dark (30 min). A mixture of acetonitrile and water was added (15 mL; 80:20, *v*/*v*), vortexed (2 min), and centrifuged (5 min, 3500 rpm). The clean-up using QuEChERS method: Extract solutions (10 mL) were added to dispersive SPE kits (150 mg PSA sorbent, 150 mg of C_18_ sorbent, and 900 mg of MgSO_4_), shaken (5 min), and centrifuged (5 min, 3500 rpm). 5 mL was collected and evaporated, then redissolved in water (200 µL). The clean-up using the SPE method: Saturated NaCl solution (4 mL) was added to the acetonitrile extract then dried under a stream of nitrogen. Phosphate solution (pH 5) was added. Final volume 30 mL. ENV+ cartridges conditioned: Methanol (2 mL), water (2 mL), and 0.05 M sodium dihydrogen phosphate solution (2 mL, pH 5). Sample was loaded and washed with phosphate solution (3 mL, pH 5) and water (1 mL). Eluted with 4 mL of acetonitrile:methanol:water mixture (45:45:10, *v*:*v*:*v*). Evaporated and redissolved in water (200 μL).	LC-MS/MS (ESI+)	Column Agilent Technologies Zorbax Eclipse XDB-C8 column (4.6 × 150 mm; 5 µm) Akady precolumn Kromasil C8 (4.6 × 15 mm; 5 µm) Mobile phase Gradient: A: 0.1% formic acid in water B: 0.1% formic acid in acetonitrile	[53]
9 β-lactam antibiotics	bovine, porcine, and chicken muscle	Samples (4 g) were homogenized, placed into a 50 mL centrifuge tube, mixed with penicillin solutions and an internal standard, and allowed to sit (15 min). Water was added (2 mL), vortexed (1 min), and acetonitrile was added (20 mL), vortexed (1 min), and centrifuged (5 min, 3500 rpm, 25°C). Saturated NaCl solution (2 mL) was added to the acetonitrile extract then dried under a stream of nitrogen. 50 mM phosphate buffer was added (25 mL, pH 5–8.5). Purification was performed (isolute ENV+, 200 mg, 3 mL), then it was conditioned (2 mL methanol, 2 mL water and 2 mL phosphate buffer pH = 5.0). The samples were loaded and subsequently washed (3 mL phosphate buffer pH = 5.0, 1 mL water), dried, and eluted (2 mL methanol and 2 mL acetonitrile). They were evaporated and redissolved (200 μL of water), then centrifuged (13,000 rpm, 5 min).	LC-MS/MS (ESI+)	Column Agilent Technologies Zorbax Eclipse XDB-C8 column (4.6 × 150 mm) Akady precolumn Kromasil C8 (4.5 × 20 mm) Mobile phase Gradient: A: 0.1% formic acid in water B: 0.1% formic acid in acetonitrile	[78]
3 β-lactam antibiotics	beef, milk	In the first step, proteins and fats were removed. Beef samples (10 g) were homogenized and mixed with acetone–acetonitrile solution (20 mL; 4:1, *v*/*v*). Milk samples (3 mL) were mixed with an acetone–acetonitrile solution (6 mL; 5:1, *v*/*v*) [28]. The samples were centrifuged (4500 rpm, 20 min) and evaporated (37 °C in a nitrogen atmosphere). Then, they were mixed with phosphate buffer (1 mL, pH 8) and 100 mmoll^−1^ tetrabutylammonium bromide (600 µL). Saturated ammonium sulfate and acetonitrile (2 mL) were added, and the volume was topped up to 10 mL with water. They were mixed and left until the phases were completely separated. The upper layer was directly injected into the HPLC.	HPLC-PDA	Column Waters Xbridge™ C18 column (250 mm × 4.6 mm, 5 µm) Mobile phase Isocratic (75:25, *v*/*v*) A: 5 mmoll^−1^ phosphate buffer (pH 6.6) B: acetonitrile	[79,80]
12 β-lactam antibiotics	milk	Conditioned Oasis HLB columns (2 mL methanol, 2 mL water). Sorbent was transferred to a glass beaker, and milk sample (500 µL), standard solution (500 µL), and half the quantity of a QuEChERS tube (125 mg) were added, mixed, and sonicated (10 min) in an ultrasonic bath. Samples were transferred to an empty cartridge reservoir, compressed, dried under vacuum, washed (5 mL of water (7% acetone)), and eluted (1 mL methanol, 2 mL of acetonitrile). They were evaporated to dryness under a nitrogen stream in a water bath (35 °C), then dissolved (500 µL water). A 20 µL aliquot was taken and injected into the HPLC.	HPLC-PDA	Column MZ-Analysentechnik Inertsil ODS-3 colum (5 µm, 250 × 4 mm) Mobile phase Gradient: A: 0.05 M ammonium acetate B: acetonitrile	[81]
8 β-lactam antibiotics	catfish	Sample (4 g) was minced and weighed in an 80 mL centrifuge tube. 20 mL acetonitrile/water (9:1) was added. Sample was homogenized. Extraction was performed in an orbital shaker (5 min). Sample was centrifuged (4000× *g*, 15 min, at 4 °C). The supernatant was filtered (nylon 0.45 µm). Filtrate (0.25 mL) was combined with McIlvaine buffer (4.75 mL, pH 7.6) and filtered with a 0.45 µm pore diameter. The online SPE system was used. C18 cartridges were conditioned with methanol (2 mL; 5 mL min^−1^ flow rate) and water (2 mL; 2 mL min^−1^). The filtrate was diluted (1:100) and inserted into the C18 cartridge (1 mL). The analytes were eluted (2.5 mL of water).	On-line SPE LC-MS/MS (ESI−)	Column Phenomenex Synergy Max column (150 × 4.6 mm; 4 μm) Mobile phase Gradient A: 2 mM ammonium formate B: 0.1% formic acid in water and acetonitrile	[82]
22 β-lactam antibiotics	poultry muscle	Samples (2.5 g) were homogenized and mixed with an internal standard (200 μL). Borate solution (10 mL) and piperidine (500 μL) were added, shaken (5 s), incubated in a water bath (1 h, 60 °C), and cooled (10 min, room temperature), and n-hexane (10 mL) was added. After shaking (5 min), the sample was centrifuged (3500× *g*, 15 min). The aqueous layer was transferred into a clean tube and neutralized (pH 7.2) using acetic acid (25%) and/or ammonia (2.5%). Phenomenex Strata-X (200 mg, 6 mL) was conditioned (5 mL methanol, 5 mL water). The samples were loaded and subsequently washed (5 mL methanol/water; 1:9, *v*/*v*), dried (5 min), eluted (5 mL methanol/acetonitrile; 50:50, *v*/*v*), evaporated (45 °C), and redissolved (500 μL 1% piperidine in water).	LC-MS/MS (ESI+)	Column Waters Acquity UPLC CSH C_18_ (2.1 × 100 mm, 1.7 μm) Mobile phase Gradient: A: 0.0032% ammonia in water B: 0.0032% ammonia in water/acetonitrile (1:9, *v*/*v*)	[68]
9 β-lactam antibiotics	ewe milk	Samples (5 mL) were homogenized and transferred into a test tube (15 mL). Acetonitrile was added (5 mL), agitated (1 min), and allowed to settle (10 min, room temperature). Then, they were centrifuged (3500 rpm, 20 min, 4 °C). The supernatant was filtered through a syringe membrane filter (0.45 μm, Millex-HN). Spe-ed SPE C18 was conditioned (5 mL acetonitrile, 5 mL of water, each for two cycles under vacuum for 15 s). The analytes retained on the cartridge were eluted (2 × 3 mL phosphate buffer (pH 3.4) and acetonitrile mixture (30:70, *v*/*v*)), evaporated, and reconstituted (500 μL of deionized water). 100 µL was taken for analysis.	HPLC-DAD	Column Scharlau Supelcosil LC 18 DB 5 µm (15 cm × 4.6 mm) Mobile phase Gradient: A: 25 mM phosphate buffer solution (pH 3.4) B: acetonitrile	[83]
14 β-lactam antibiotics	bovine milk	Sample was weighed (2 mL) and placed into a centrifuge tube (50 mL). Analytes were extracted with 4 mL of acetonitrile, added in three steps (2 mL, 2 mL, 1 mL), and manually mixed. Analytes were kept in a horizontal mixer (15 min). Tubes were centrifuged (5 min at 4000× *g*; 5 °C). Supernatant was transferred to a tube (50 mL) containing C18 bulk (at least 100 mg). Tubes were mixed with a vortex (15 s) and centrifuged (5 min at 4000× *g*). Supernatant was transferred to a clean tube (15 mL), kept in a freezer (−17 °C, 20 min). Tubes were centrifuged (10 min at 4000× *g*, 0 °C). Supernatant was transferred to a 50 mL tube and evaporated (water bath, ≤45 °C) with a N_2_ stream. Volume of the solvent was reduced to ~500 µL. The final amount was −1 mL. Extract was transferred into a HPLC vial.	LC-MS/MS (ESI+)	Column Angela Technologies DuraShell RP column (C18, 100 × 2.1 mm, 3 µm, 150 Å) Phenomenex SecurityGuard Cartridge C18 (4 × 3.0 mm) Mobile phase Gradient: A: 0.1% formic acid B: 0.1% formic acid in methanol	[40]
14 β-lactam antibiotics	cow milk	Samples of milk underwent freeze-drying. Samples (1 g) were mixed with standard solution. McIlvaine buffer (3 mL, pH 6.0) was added and vortexed (1 min). An acetonitrile:methanol mixture (12 mL, 70:30; *v*/*v*) was added, vortexed (2 min), sonicated (20 min), and centrifuged (3 min at 4109× *g*). Supernatant was transferred to a tube containing 300 mg of dispersive sorbent (PSA) and 900 mg of anhydrous MgSO_4,_ shaken (1 min), and centrifuged (1 min, 4109× *g*). It was evaporated (40 °C) and redissolved in 1 mL of a solution of ammonium formate (50 mM, pH 4.0) and methanol (80:20; *v*/*v*). It was then centrifuged (15 min; 16,300× *g*) and injected into the LC system.	UHPLC-MS/MS (ESI+)	Column Waters Acquity UPLC BEH^TM^ C18 column (1.7 µm; 2.1 × 100 mm) Mobile phase Gradient: A: ammonium formate 50 mM, pH 4.0 B: methanol	[84]
6 β-lactam antibiotics	milk	Samples of milk (30 g) were mixed with standard solution and centrifuged (30 min, 10,800× *g*, 35 °C). 1 g of the defatted milk (middle fraction) was taken and diluted to 10 mL (0.05 M phosphate buffer, pH 7.5). MIPs (20 mg) were loaded to solid-phase extraction cartridges. Cartridges were equilibrated (10 mL of methanol and 10 mL of 0.05 M phosphate buffer at pH 7.5). Samples (10 mL) were applied and passed through the cartridges (steady flow rate of 0.50 mL/min using a peristaltic pump). Cartridges were rinsed (5 mL of a mixture of methanol and 0.1 M HEPES buffer (pH 7.5, 2:98 *v*/*v*)). The analytes were eluted (1 mL of the mixture of methanol containing 0.1% trifluoroacetic acid). The eluate (200 µL) was taken and diluted (800 µL of water) and injected into the UHPLC-MS/MS system for analysis.	UHPLC-MS/MS (ESI+)	Column Waters Acquity UPLC BEH^TM^ C18 column (1.7 µm; 1.0 × 150 mm) Mobile phase Isocratic: water:acetonitrile:formic acid (74.9:25.0:0.1, *v*/*v*/*v*)	[70]
16 β-lactam antibiotics	pork muscle	Sample (2 g) was put into a glass grinder. The dispersion absorbent (Oasis HLB) was preconditioned (10 mL acetonitrile, distilled water, 5 mL 2% NaCl solution). The sample was blended with the dispersion absorbent (3 g) and put into a glass column. The column was compressed and washed (6 mL of n-hexane). Analytes were eluted (8 mL acetonitrile and water, both containing 0.1% formic acid; 50:50). Ethyl acetate (6 mL) was added and mixed (1 min), then centrifuged (3000× *g*; 10 min). Dispersion was repeated twice. The supernatant was dried (nitrogen dryer, 50 °C). Residue was dissolved (2 mL, acetonitrile/water; 10:90, *v*/*v*).	UPLC-MS/MS (ESI+)	Column ACQUITY UPLC HSS T3 C_18_ column (1.7 mm, 2.1 × 100 mm) Mobile phase Gradient: A: 0.1% formic acid in water B: 0.1% formic acid in acetonitrile	[85]
17 β-lactam antibiotics	muscles	Sample (0.5 g) was weighed into a tube. 0.1 M of EDTA (100 μL) was added. The muscle was extracted with acetonitrile and water (3 mL, 80/20, *v*/*v*). The sample was shaken and centrifuged. A second extraction was performed with acetonitrile (3 mL). Collected extracts were evaporated and redissolved (1.5 mL ammonium acetate 0.2 M). Ultracentrifugation was performed. Sample was injected.	LC-HRMS/MS ESI+	Column Agilent Technologies Poroshell 120 EC-C18 column (100 × 3.0 mm; 2.7 μm) Poroshell (2.1 × 5 mm) guard column Mobile phase Gradient: A: aquenous solution with 0.1% (*v*/*v*) formic acid B: methanol	[86]
30 β-lactam antibiotics	bovine muscles	Samples were weighed (2 g ± 0.01 g) into a polypropylene centrifuge tube (50 mL). Internal standard solution (100 μL) and working standard solution were added. The samples were left for 15 min. Water (1.9 mL) and acetonitrile (8 mL) were added. The samples were homogenized (20 s) and centrifuged (15 min; 2842× *g*, 4°C). The supernatant was transferred to a polypropylene tube containing 500 mg of C_18_ sorbent, vortexed (40 s), centrifuged (15 min, 2842× *g*, 4°C), and evaporated under nitrogen (TurboVap, 40 °C). The final volume was filled up with water to 1 mL. The extracts were vortexed (10 s) and filtered (0.2 μm PTFE syringe filters). The extracts were transferred into autosampler vials.	UHPLC-MS/MS ESI+	Column CHS C_18_ column (2.1 × 100 mm, 1.7 µm) Mobile phase Gradient: A: 0.01% formic acid and 0.2 mM ammonium acetate in water B: 0.01% formic acid in acetonitrile	[41]
16 β-lactam antibiotics	chicken muscle	Sample (2 g) was weighed, added to a glass tuber, and mixed. Then, tissues were placed in QuEChERS extraction tubes, and acetonitrile containing 0.1% formic acid (15 mL) was added, shaken, and centrifuged (3000× *g*, 10 min). An acetonitrile layer was placed into a QuEChERS clean-up tube, shaken, and centrifuged (3000× *g*, 5 min). Obtained supernatant was put into a glass tube and dried using nitrogen, then it was reconstituted (2 mL of acetonitrile/water; 10:90, *v*/*v*). Obtained solution was filtered through a 0.22 μm nylon membrane and analyzed.	UPLC-Q-Orbitrap-MS (ESI+)	Column Waters Corporation HSS T3 C_18_ column (1.7 μm, 2.1 × 100 mm) Mobile phase Gradient: A: 0.1% formic acid in water B: 0.1% formic acid in acetonitrile	[52]
8 β-lactam antibiotics	fish, shrimp, and eel	Tissue (2.0 g) was placed into a tube. Spiking standard mixes were added and left to stand (5 min). Tissue was extracted (8 mL of 0.2% p-toluenesulfonic acid monohydrate and 2% glacial acetic acid in acetonitrile), vortexed (30 min, 2500 rpm), and centrifuged (7 min, 4 °C (min. 17,000 RCF (*g*)). The extract (3 mL) was transferred to an Oasis PRiME HLB 6 cc (200 mg) extraction cartridge. The samples were drained through the cartridges. The remaining extract was evaporated to near dryness (N_2_, 55 °C). The extract was reconstituted (400 μL of 10% acetonitrile in water (*v*/*v*)), mixed, and centrifuged (min. 28,900 RCF (g), 7 min). 300 μL of the extract was erased and placed into a LC vial.	LC-Q-Orbitrap HRMS (HESI+)	Column Supelco Ascentis Express C18 (7.5 cm × 2.1 mm, 2.7 μm) column Mobile phase Gradient: A: 0.1% formic acid in water B: acetonitrile	[64]
7 β-lactam antibiotics	heavy pigs’ urine and muscle	Urine: Samples (5 mL) were centrifuged (2500× *g*, 4°C, 5 min) and spiked. Compounds were extracted under vacuum (Oasis HLB cartridges; 3 mL, 60 mg). The cartridges were preconditioned (3 mL methanol, 3 mL 0.5 M HCI, 3 mL water). The samples were loaded. The cartridges were washed (3 mL water; 3 mL methanol:water, 20:80, *v*/*v*). The analytes were eluted (5 mL methanol). The eluate was evaporated. The extract was reconstituted (200 μL methanol:water, 10:90, *v*/*v*). Muscle: Samples (1 g) were spiked. The analytes were extracted (5 mL Mcllvaine buffer, pH 4.0). Trichloroacetic acid (100 μL, 20% *w*/*v*) was added. The samples were vortexed, sonicated (10 min), then centrifuged (2500× *g*, 4 °C, 10 min). The supernatant was transferred to a centrifuge tube and defatted (2 × 3 mL n-hexane). The n-hexane layer was removed after each centrifugation (2500× *g*, 5 min). The samples were purified and extracted by vacuum with Oasis HLB cartridges. The cartridges were preconditioned (3 mL methanol, 3 mL water). The samples were loaded. The cartridges were washed (2 × 3 mL methanol:water, 5:95, *v*/*v*). The analytes were eluted (5 mL methanol), transferred to a polypropylene tube, and evaporated. The extract was reconstituted (200 μL methanol:water, 10:90, *v*/*v*).	HPLC-MS/MS (ESI+/−)	Column Synergi Hydro-RP column (150 × 2.0 mm, 4 μm) Phonomenex C18 guard column (4 × 3.0 mm) Mobile phase Gradient: A: 0.1% aqueous formic acid B: methanol	[61]
23 β-lactam antibiotics	foods of animal origins: eggs, raw milk, processed dairy ingredients, infant formula, and meat- and fish-based products, including baby food	Samples (1 g) were weighed into polypropylene tubes (50 mL). Phosphate buffer (15 mL, 500 mm, pH 9.2) was added. Samples were homogenized with a ceramic blender. Tubes were mechanically shaken (3 min). Acetonitrile (30 mL) was added. Tubes were shaken again (1.5 min). QuEChERS salt mixtures were added. Tubes were shaken (3 min). The samples were centrifuged (10 min, 4000× *g*, RT). The supernatants (8 mL) were transferred to 15 mL polypropylene tubes containing dispersive SPE sorbents. The tubes were shaken (1.5 min) and centrifuged (4000× *g*, 5 min, RT). The supernatants (5 mL) were evaporated under nitrogen (35 ± 2 °C). Obtained volume: 0.5 mL. Water (0.6 mL) was added. The mixture was evaporated. Final volume: 0.5 mL. Extracts were transferred to polypropylene tubes and centrifuged (17,000× *g*, 10 min, 4 °C), then filtered through a 0.45 µm PTFE filter. Further analysis was performed using the LC-MS/MS method.	LC-MS/MS (ESI+/−)	Column Waters Acquity BEH VanGuard precolumn (2.1 × 5 mm, 1.7 μm) Waters Acquity BEH C18 column (2.1 × 100 mm, 1.7 μm) Mobile phase Gradient: A: 0.5 mM ammonium formate and 0.1% formic acid in water B: 0.5 mM ammonium formate and 0.1% formic acid in methanol	[54]
5 β-lactam antibiotics	fish	Homogenized samples (2 g) were weighed into a centrifuge tube. Internal standard (200 μL) and deionized water (800 μL) were added. The samples were vortexed (30 s) and left at room temperature (10 min). 5% trichloroacetic acid (8 mL) was added. The samples were homogenized (20 s) in ultra-turrax, placed in a shaker (10 min), and centrifuged (2700× *g*, 4 °C, 12 min). The extract was filtered (Millipore PVDF membrane, 4 μm pore size) before LC-MS/MS analysis.	LC-MS/MS (ESI+)	Column Agilent Technologies Zorbax Eclipse XDB C18 (150 × 4.6 mm, 1.8 μm) column Mobile phase Gradient: A: 0.1% heptafluorobutyric acid in water B: acetonitrile	[39,42]
8 β-lactam antibiotics	raw milk	SPE sorbent material was preconditioned with water (2 mL) and methanol (2 mL), and transferred into a glass beaker. Milk (500 μg) and a standard solution of the antibiotics (500 μL) with 125 mg of a QuEChERS tube were added. Homogenization with sonication in an ultrasound bath was performed (10 min). Samples were transferred to an empty cartridge and compressed. The sorbent bed was cleaned (5 mL water, 1% acetone) twice. The analytes were eluted (2 mL methanol). Samples were filtered (PVDF Durapore syringe filters; 13 mm × 0.45 μm) and evaporated to dryness (nitrogen stream). The residues were dissolved (500 μL water). The obtained samples (100 μL) were transferred to the HPLC system.	HPLC-DAD	Column MZ-Analysentechnik Perfectsil ODS-2 (5 μm, 250 × 4 mm) Mobile phase Gradient: A: 0.05 M CH_3_COONH_4_ B: acetonitrile	[87]
11 β-lactam antibiotics	bovine tissues: kidney, liver, and muscle	Samples (2 g) were weighed. Acetonitrile/water (4/1; *v*/*v*; 10 mL) was added. The solution was placed in a shaker (5 min). The samples were centrifuged (4150 rpm, 3 min, room temperature). 407 μL of the extract was placed into autosampler vial. Aqueous 146.5 mM 1-heptanesulfonate reagent solution (273 μL) was added. 7/3 (*v*/*v*) acetonitrile/water (50 μL) was added.	LC-MS/MS (ESI+/−)	Column Waters Acquity HSS T3 analytical column (10 × 2.1, 1.8 μm) Waters Vanguard column (0.5 cm) Mobile phase Gradient: A: 0.1% formic acid in water B: 0.1% formic acid in acetonitrile: methanol 1:1 (*v*:*v*)	[88]
17 β-lactam antibiotics	muscle and milk	Samples (1.50 g) were weighed into a Falcon tube. 0.1 M of EDTA (100 μL) was added to muscle samples. The samples were extracted with acetonitrile/water (3 mL, 80/20, *v*/*v*). The milk samples were extracted with 0.1 M of EDTA (1 mL) and acetonitrile (3 mL). Both matrices were extracted for the second time with acetonitrile (3 mL). The samples were centrifuged. The extracts were evaporated and solubilized (1.5 mL, ammonium acetate 0.2 M) and injected into the LC system (10 μL).	LC-MC/MC (ESI+)	Column Agilent Technologies Poroshell 120 EC-C18 column (3.0 × 100 mm; 2.7 μL) Agilent Technologies Poroshell guard column (2.1 × 5 mm) Mobile phase Gradient: A: aqueous solution 0.1% (*v*/*v*) formic acid B: methanol	[86,89,90]
15 β-lactam antibiotics	chicken feathers	Samples (0.2 g) were placed into a polypropylene tube. Internal standards were added. 0.02 M oxalic acid (1 mL, pH 4) was added. The samples were vortexed (3 min). 0.1 M Na_2_EDTA (0.5 mL) was added. The samples were mixed (3 min). Acetonitrile (8 mL) was added, shaken on a rotary tumbler (30 min), and centrifuged (10 min, 4200× *g*). The supernatants were put into Oasis HLB cartridges. Extracts were evaporated to dryness (N_2_, 40 °C). The residue was redissolved (0.025% HFBA; 0.5 mL). The samples were filtered (PVDF filter; 0.22 µm). UHPLC-MS/MS analysis was performed.	UHPLC-MS/MS (ESI+)	Column Agilent Technologies ZORBAX SB-C18 (50 mm × 2.1 mm × 1.8 μm) Phenomenex octadecyl guard column (2 × 4 mm) Mobile phase Gradient: A: acetonitrile B: 0.025% HFBA	[63]
32 β-lactam antibiotics	milk	Samples (2 g) were weighed into polypropylene tubes. Standard solution was added, mixed, and left to stand for 15 min. Water (1 mL) and acetonitrile (7 mL) were added, then vortexed for 1 min. Samples were centrifuged (15 min, 2842× *g*, 4°C). Supernatant was transferred to a 50 mL d-SPE polypropylene tube containing 500 mg endcapped C18 sorbent, vortexed (40 s), and centrifuged (15 min, 2842× *g*, 4 °C). Supernatant was transferred (15 mL tube), evaporated in a TurboVap (under nitrogen, 40°C) to <1 mL, and adjusted to 1 mL with water. Extracts were vortexed (10 s) and centrifuged (15 min, 2842× *g*, 4 °C). The extract (400 µL) was filtered through syringeless Mini-UniPrep PTFE devices and injected into the UHPLC-MS/MS system for analysis.	UHPLC-MS/MS (ESI+)	Column Agilent Zorbax Eclipse Plus Phenyl-Hexyl Rapid Resolution HD analytical column (3.0 × 100 mm, 1.8 μm) fitted with an in-line filter (0.2 μm) Mobile phase Gradient: A: 0.01% formic acid with 0.2 mM ammonium acetate in water B: 0.01% formic acid in acetonitrile	[91]
19 β-lactam antibiotics	meat, kidneys, liver, bacon, milk, and eggs honey	Option I: Sample (1 g), acetonitrile (2 g), NaCl (0.5 g), and EDTA (40 mg) were put into a centrifuge tube (15 mL). The samples were stirred (5 min) and centrifuged (5 min at 2700 rpm). The acetonitrile layer was collected and evaporated to dryness (40 °C) under nitrogen. Methanol (50 μL) and water (950 μL) were added to the residue, stirred (5 min), and filtered through a membrane filter for chromatography. Option II: Honey sample (1 g) was dissolved in water (1 mL). The sample, succinic acid (12 mg), EDTA (40 mg), and water (2 mL) were put into a centrifuge tube (15 mL) and stirred manually. Acetonitrile (2 mL) and ammonium sulfate (2 mg) were added, stirred (5 min), and centrifuged (5 min at 2700 rpm). The acetonitrile layer was collected and evaporated to dryness (40 °C) under nitrogen. Methanol (50 μL) and water (950 μL) were added to the residue, stirred (5 min), and filtered through a membrane filter for chromatography.	UHPLC-Q-TOF	Column Waters Acquity UPLC^®^BEN C18 (30 × 2.1 mm) Mobile phase Gradient: A: 0.1% formic acid in water B: 0.1% formic acid in acetonitrile	[92]
1 β-lactam **antibiotic**	milk bovine, porcine, and chicken muscle	Samples of milk (10 g) were mixed with a standard solution, and acid-acetonitrile (4 mL, pH = 4.0) was added and centrifuged (10 min, 3000 rpm). Supernatant was evaporated and dissolved (10 mL of water). Muscle samples of chicken, pork, and beef (5 g) were mixed with a standard solution, and acetonitrile (3 mL) was added. Samples were mixed and centrifuged (10 min, 3000 rpm). Supernatants were evaporated and dissolved (10 mL of water). MIPs (120 mg) were packed into solid-phase extraction cartridges. MISPE cartridges were conditioned (10 mL methanol, 10 mL water). Prepared aqueous sample solutions (10 mL) were loaded onto the cartridges and eluted (1.5 mL, methanol:water 70:30, *v*/*v*). Eluents were analyzed using HPLC-UV.	HPLC-UV	Column C18 column (4.6 × 150 mm) Mobile phase Isocratic: acetonitrile:water contained 0.2% acetic acid (30/70, *v*/*v*)	[71]
16 β-lactam antibiotics	eggs	Samples (1.5 g) were weighed into a polypropylene tube. The samples were spiked. 0.1 M Na_2_EDTA (0.5 mL) and acetonitrile:H_2_O 4:1 (*v*/*v*; 3 mL) with 0.05% formic acid were added, shaken, and centrifuged. The supernatant was transferred to a polypropylene tube. Extraction was repeated again with acetonitrile (3 mL), shaken, centrifuged, and sonicated (10 min). Supernatants were reunited and evaporated (40 °C; N_2_). Resuspension (1.5 mL ammonium acetate 0.2 M) and centrifugation (14,000 g, 30 min, 4 °C) were performed. 5 μL was injected into the LC-HRMS system.	LC-HRMS	Column Agilent Technologies Poroshell 120 EC-C18 32k (100 × 3 mm; 2.7 μm) Agilent Technologies Poroshell guard column (2.1 × 5 mm) Mobile phase [86] Gradient: A: aqueous solution with 0.1% (*v*/*v*) formic acid B: methanol	[93]
4 β-lactam antibiotics	cereals, meat, eggs, milk, vegetables, and fruits	Samples were weighed in a polypropylene centrifuge tube (1.0 ± 0.1 g dry weigh) and spiked. The samples were ultrasonically extracted (10 mL acetonitrile:water (4:1, *v*/*v*) with 0.1% formic acid, 10 min). The sampes were extracted by a vertical oscillator (30 min) and centrifuged (9000 rpm, 10 min, 3 ± 1°C). Second extraction: DisQue salt pack and 10 mL of acetonitrile:water (4:1, *v*/*v*) with 0.2% formic acid. Third extraction: 10 mL of acetonitrile:water (4:1, *v*/*v*) with 0.2% formic acid. Oasis PRiME HLB cartridge was preconditioned (10 mL of methanol). Extracts were concentrated to 6 mL and transferred to the cartridge (5–10 mL/min). The eluate was collected, evaporated to dryness (nitrogen, Turbovap, 35 °C), and reconstituted with methanol (0.5 mL) in a glass vial (2 mL), then stored at −20 °C until analyzed.	UHPLC-MS/MS (ESI+)	Column Waters BEH C8 column (1.7 μm × 2.1 × 100 mm) Mobile phase Gradient: A: 0.1% formic acid in water B: methanol	[65]
14 β-lactam antibiotics	milk: cow, sheep, and goat	Samples (1 g) were placed into a polypropylene tube (25 mL). EDTA 0.1 M (100 μL) and ACN with 2% formic acid (4 mL) were added. The samples were vortexed (30 s) and centrifuged (6000 rpm, 5 °C, 10 min). Supernatant was loaded on an Oasis HLB PRiME cartridge, which had been preconditioned (3 mL, acetonitrile). Purified extract (100 μL) was placed into a vial, diluted (900 μL solution of ammonium acetate 0.2 M:methanol 9:1 (*v*/*v*)), and analyzed.	LC-Orbitrap-HRMS	Column Agilent Technologies Poroshell 120 EC-C18 (100 × 3 mm; 2.7 μm) Agilent Technologies Poroshell guard column (2.1 × 5 mm) Mobile phase Gradient: A: 0.1% formic acid in water B: methanol	[66]
3 β-lactam antibiotics	chicken tissue	Samples (2 g) were placed into a polypropylene centrifuge tube (50 mL), spiked, shaken manually (1 min), and left at a room temperature (20 min). Extraction solvent (10 mL, acetonitrile/methanol (10:20, *v*/*v*)) was added. The mixture was vortexed (1000 rpm, 1 min), sonicated (15 min), and centrifuged (3500 rpm, 5 min). Top supernatant was transferred to a flask (1 mL) through a syringe filter (0.2 μm, nylon) and evaporated to dryness (35°C, rotary evaporator). The residues were redissolved with methanol (1 mL). Residue (20 μL) was injected into the LC-UV system.	LC-UV	Column Phenomenex Hypersil BDS-C18 (3 μm, 100 mm × 4 mm) Mobile phase Isocratic 0.05 M Na_2_HPO_4_:acetonitrile:methanol (70:10:20), pH 8	[94]
52 β-lactam antibiotics	meat and poultry, aquatic products, milk, and eggs	Samples (1 g) were placed into a 50 mL polypropylene centrifuge tube, and acetonitrile:water mixture (12 mL, 75:25, *v*/*v*) was added and vortexed (3 min). Solution was centrifuged (5 min, 10,000 rpm, 4 °C), and supernatant (9 mL) was transferred into a 15 mL polypropylene tube containing 300 mg C18 sorbent. Samples were vortexed (30 s) and centrifuged (5 min, 10,000 rpm, 4 °C). Supernatant (6 mL) was transferred to a 15 mL polypropylene tube and evaporated (under a nitrogen stream, 40 °C). Residue was dissolved (1 mL, acetonitrile:0.1% formic acid (1:9, *v*/*v*)). Extract was vortexed (10 s) and filtered (0.2 µm PTFE syringe filter). Extract was injected into the UPLC-MS/MS system.	UPLC-MS/MS (ESI+)	Column Agilent ZORBAX SB-Aq column (2.1 × 150 mm, 3.5 µm) Mobile phase Gradient: A: 0.4% formic acid in water B: acetonitrile	[95]
